# Retraction Note: Association of milk consumption with the incidence of cholelithiasis disease in the US adult population

**DOI:** 10.1186/s12889-024-19943-3

**Published:** 2024-09-05

**Authors:** Yu Ma, Yahui Liu, Feng Jia

**Affiliations:** Department of Hepatobiliary and Pancreatic Surgery, General Surgery Center, the First Hospital of Jilin University, Changchun, Jilin People’s Republic of China

**BMC Public Health (2023) 23:1639**

10.1186/s12889-023-16615-6

The authors have retracted this article. After publication, they were alerted to an error in their calculation: some participants in the NHANES data releases for 2017–2020, 2019–2020, and 2017–2018 were inadvertently double counted, leading to an inflated figure of 24,814 rather than 15,560. The authors are preparing a new manuscript for submission and peer review. All authors agree with this retraction.

